# Changes in flavor volatile composition of oolong tea after panning during tea processing

**DOI:** 10.1002/fsn3.307

**Published:** 2015-11-01

**Authors:** Ershad Sheibani, Susan E. Duncan, David D. Kuhn, Andrea M. Dietrich, Jordan J. Newkirk, Sean F. O'Keefe

**Affiliations:** ^1^ Department of Food Science and TechnologyVirginia TechBlacksburgVirginia24061; ^2^ Department of Civil and Environmental EngineeringVirginia TechBlacksburgVirginia24061

**Keywords:** Flavor analysis, GC‐MS, GC‐O, oolong tea, panning

## Abstract

Panning is a processing step used in manufacturing of some varieties of oolong tea. There is limited information available on effects of panning on oolong tea flavors. The goal of this study was to determine effects of panning on flavor volatile compositions of oolong using Gas Chromatography‐Mass Spectrometry (GC‐MS) and Gas Chromatography‐Olfactometry (GC‐O). SDE and SPME techniques were applied for extraction of volatiles in panned and unpanned teas. A total of 190 volatiles were identified from SDE and SPME extractions using GC‐MS and GC‐O. There were no significant differences (*P* > 0.05) in aldehyde or terpene contents of unpanned and panned tea. However, alcohols, ketones, acids and esters contents were significantly reduced by panning. Among 12 major volatiles previously used for identification and quality assessment of oolong tea, *trans* nerolidol, 2‐ hexenal, benzaldehyde, indole, gernaiol, and benzenacetaldehyde contents were significantly decreased (*P* < 0.05) by panning. Panning increased (*P* < 0.05) contents of linalool oxide, *cis* jasmone, and methyl salicylate. The GC‐O study also showed an increase of aroma active compounds with sweet descriptions and decrease of aroma active compounds with fruity and smoky descriptions after panning. Panning significantly changes the volatile compositions of the tea and created new aroma active compounds. Results from this study can be used in quality assessment of panned oolong tea.

## Introduction

Oolong tea is manufactured predominantly in southeast China and Taiwan (Lee et al. 2008). Less than 2% of tea manufactured in the world is semi‐fermented oolong tea (Hara et al. 1995); however, due to the complex processing steps and the limited supply, oolong teas usually have a higher unit price than green or black teas in the international tea market. Current increases in oolong tea consumption might result from the recent studies on health benefits of tea polyphenols, and also the unique taste and aroma of this tea variety.

Oolong tea categorized as a semi‐fermented tea. For tea, fermentation refers to the natural browning reactions induced by oxidative enzymes in the cells of tea leaves. This process is mainly the oxidative polymerization of catechins catalyzed by polyphenol oxidase and peroxidase (Chaturvedula and Prakash 2011). Oolong tea is generally fermented from about 10% to 60% to create a taste and color somewhere between green and black teas. Fermentation is responsible for creation of many flavor compounds. During the fermentation process, the tea leaves are injured and, consequently, an increase in enzyme activity is seen with the creation of aromatic alcohols (Ma et al. 2014). Wang et al. (2008) found that fermentation can cause the loss of grassy or green flavors and the formation of fruity/floral flavors, providing much of black tea's sweet and bold flavor. There are no standard recipes or procedures on how to manufacture oolong tea. The processing and the level of oxidation are decided by each tea garden or tea master. In Taiwan, Baozhong tea would typically be fermented around 10%, ball rolled Donding oolong from 15–25%, and Baihao oolong (Oriental Beauty) around 60–80%.

Panning is a processing step in the manufacturing of some varieties of oolong tea that is performed after fermentation. There are two steps often called panning: first, exposure of tea leaves to heat in order to inhibit fermentation by inactivating the enzymes, and second, heating after drying to develop unique flavors in oolong tea (Hui et al. 2003; Zhen 2003; Info Taiwan 2014). During this first process, tea leaves lose moisture and thus are softer making the rolling into string shapes and dehydration easier (Hui et al. 2003). Panning can be done using rotary pan or panning machine, convection oven, or via pan‐frying. The exact temperature and time depends on various teas and determined by tea masters. The panning period also depends on the variety of tea leaf and loading quantity. Panning is known to eliminate grassy odor while leaving a nutty smell and taste. Panning also prevents tea leaves from breaking before they are rolled and the result is soft and flexible leaf texture with a strong pleasant aroma (Info Taiwan 2014). However, there is limited information available on the effects of panning after drying on volatile compounds of oolong tea. In addition, the compounds responsible for the aroma of panned tea still need clarification.

Flavor analysis of oolong tea is important for variety authentication and quality assessment. Oolong varieties are sold at a premium price compared to lower‐grade varieties. Some oolong varieties are very similar in appearance and flavor and accurate identification and differentiation is only possible for tea experts or experienced tea tasters (Zhang et al. 2013). Thus, there is significant interest in developing accurate chemical methods for quality assessment and identification of oolong tea varieties. Composition of the volatile compounds extracted by extraction methods SDE and SPME may differ, and it is advisable that two methods used together for effective volatile analysis (Thompson‐Witrick et al. 2015).

Nonvolatile components are generally responsible for taste, while volatile components give the aroma (Rawat et al. 2007). The unusual taste of oolong tea infusion depends on the degree of fermentation, the elevation and growing conditions as well as the tea bush. Nonaka et al. (1983) (as cited in Chaturvedula and Prakash 2011) reported the fruity and sweet taste of oolong tea infusion are the integrated taste of nonoxidized catechins, thearubigins, some secondary polyphenolic compounds, caffeine, free amino acids, sugars, and volatile compounds. Compared to green tea, the astringency of oolong tea is lower and the sweetness is higher. Volatile flavor compounds of tea are mostly composed of nonterpenoids or terpenoids, which are responsible for sweet flowery aroma of tea (Rawat et al. 2007). Previous studies showed volatile compounds such as *trans*‐nerolidol, *trans*‐2‐hexenal, benzaldehyde, methyl‐5‐hepten‐2‐one, methyl salicylate, indole (Wang et al. 2008; Pripdeevech and Machan 2011), *cis*‐jasmone (Pripdeevech and Machan 2011), (E)‐geraniol, (E)‐*β*‐damascenone, linalool oxide B, benzaldehyde (Kawakami et al. 1995; Wang et al. 2008; Wang et al. 2011; Zhang et al. 2013), (E,E)‐2,4‐heptadienal and (Z)‐3‐hexenol (Wang et al. 2008, 2011) are the key odorants, and indicators of high quality oolong tea.

The objectives of this study were to investigate effects of panning on flavor volatile compositions of oolong tea and to determine changes in aroma active compounds of panned compared to unpanned oolong tea using Gas Chromatography‐Mass Spectrometry (GC‐MS) and Gas Chromatography‐Olfactometry (GC‐O).

## Materials and Methods

### Panning process

Three batches of unpanned Jin Xuan (*Chin*‐*Hsuan*, or Zhu Shan) oolong tea samples were purchased from Tea of Life^®^ Health Inc. in Rosedale, NY before the experiment and stored at room temperature. To pan the tea leaves, 680 grams of oolong tea was placed on a metal baking dish. Then, the dish was heated/panned in a convection oven at 120°C for 6 h. The method and selected condition for the panning process was based on our preliminary study and literature. After heating, the tea leaves were cooled to room temperature. The panning and flavor analysis were performed for the three batches separately.

### Volatile extraction using SDE

In preliminary experiments, we optimized conditions for SDE extraction including solvent and extraction time for flavor analysis of tea, and we applied the optimal extraction condition to this study. A simultaneous distillation, extraction (SDE), Likens‐Nickerson, apparatus was used. Tea leaves (50 g) were placed in a 1 L round bottom flask containing 400 mL of distilled water. One hundred milliliter of HPLC grade diethyl ether (Sigma‐Aldrich Co., St. Louis, MO) with 0.5 mL of 100 ppm ethyl decanoate (internal standard) (Sigma‐Aldrich Co.) was placed into a 250 mL extraction flask. Two electric heating plates were used to maintain boiling for the tea and solvents in the SDE apparatus. The volatiles were steam‐distilled and extracted into diethyl ether for 40 min. After extraction, the solvent was dried over anhydrous sodium sulfate (Fisher Scientific, Pittsburg, PA) and filtered. Then, the extract was concentrated to 2 mL in a vacuum rotary evaporator and nitrogen gas. The concentrates were analysed using Gas Chromatography – Mass spectrometry (GC‐MS) and Gas Chromatography Flame Ionization Detection – Olfactometry (GC‐ FID/O) for volatile analysis.

### Volatile extraction by SPME for GC‐MS analysis

Four grams of tea leaves were placed in 200 mL of hot distilled water (98°C) and brewed for 5 min. Then, 5 mL of the filtered tea infusion and 1 g of NaCl were placed into 10 mL headspace vials with Teflon‐lined silicon septa (Chromacol, Fisher Scientific). SPME was used to extract volatiles and volatiles were analysed by injection into the GC‐MS using an AOC‐5000 Plus (Shimadzu Scientific, Columbia, MD) SPME auto‐sampler. Samples were equilibrated for 2 min prior to extraction. A DVM/Carboxen/PDMS SPME fiber (2 cm 50/30 um) (Supelco, Bellefonte, PA) was exposed to the headspace above the tea extract in glass vials for 30 min at 40°C with an agitation speed of 250 rpm.

### Volatile extraction by SPME for GC‐FID/GC‐O analysis

The extraction and injection were performed manually for GC‐O analysis. Five milliliter of tea aqueous infusions (which were prepared similar to GC‐MS analysis) was placed in a 15 mL amber glass vial with a Teflon‐lined cap. A “RTC basic” heater with an ETS D4 Fuzzy Logic Controller (IKA Werke, Wilmington, NC) was used to heat samples at 40°C while being stirred using a 4 mm stir bar. A 50/30 *μ*m SPME fiber (DVB/CAR/PDMS) on a 2 cm StableFlex fiber (Supelco Bellefonte, PA) was inserted into the vial and was exposed approximately 1 cm above the headspace for 30 min while a magnetic bar continued to stir the sample.

### GC‐MS analysis

The volatile constituents of each sample were analyzed using a Shimadzu GCMS‐QP2010 Ultra gas chromatograph with mass selective detector (Shimadzu) equipped with GCMSsolutions 2.53 and capillary nonpolar column (SHRXI‐5MS, Shimadzu, 30 m * 0.25 mm id * 0.25 *μ*m film thickness). The oven temperature was initially held at 50°C for 5 min and then increased at 4º C/min to final temperature of 250°C. The injector temperature was 200°C and injections were made in splitless mode. Ultra high purity helium used as a carrier gas at a flow rate of 0.69 mL/min (approximately 25 cm/sec linear flow velocity). The mass spectra were collected at m/z 40–400 and were performed every 0.3 sec. The ion source and quadrupole were set at 230 and 200°C respectively. Identification of the volatile components was performed by combined matching standardized retention time (LRI values) for a DB‐5 column (Flavornet and Pherobase) and fragmentation spectra of standards from NIST 11 (Scientific Instrument Services, Ringoes, NJ) and the Wiley 2010 libraries (John Wiley and Sons Inc.). Confirmation of the identification was sought by matching the mass spectra of the compounds with the reference mass spectra present in the NIST 11 and Wiley libraries. The results were compared with our control, unpanned samples.

### GC‐O analysis

Approval for use of human subjects in research was obtained from the Virginia Tech Institutional Review Board before GC‐O experiments began (IRB #13‐580). GC‐O analysis was carried out using a HP 5890A gas chromatograph (Hewlett‐Packard Co., Palo Alto, CA) equipped with a flame ionization detector (FID), a sniffing port (ODOII; SGE Inc. Austin, TX), and a DB‐5 ms column (30 m × 0.25‐mm i.d. × 0.25 *μ*m film thickness) (J&W Scientific, Folson, CA). The detector and injector were set to 250°C and 275°C, respectively; all injections were made in the splitless mode. The initial oven temperature was 50°C and increased at 10°C/min until reaching a final temperature of 200°C. Chromatograms were recorded using a HP 3396A integrator (Hewlett‐Packard Co., Palo Alto, CA). Hydrogen was used as the carrier gas with a flow rate of 1.0 mL min^−1^ (linear flow velocity ~ 25 cm/sec). The GC column effluent was split 1:1 between the FID and the ODOII using deactivated fused silica capillaries (1‐m length × 0.32 *μ*m i.d.). Two trained assessors were selected for GC‐O analysis. The assessors sniffed tea extracts from SDE or SPME methods for 20 min from each batch. Aroma descriptions, times and intensity were recorded for every sample. The assessors indicated aroma intensity in scale 1–5 where 1 was the lowest intensity and 5 was the highest.

Mean aroma intensities for each odorant were calculated by averaging the reported intensity by panelists. Aroma‐active compounds were defined as the ones that were detected by the panelists fifty percent of the time with similar descriptions and retention times or those scored higher than 3 by panelists. Kovats or Linear Retention Index (LRI) values were determined using a series of alkanes (C5–C26) which were run under identical conditions. Identification of volatile compounds was based upon their odor descriptions and RI values from DB‐5 column. The databases Flavornet (http://www.flavornet.org/flavornet.html) and Pherobase (http://www.pherobase.com/) were used to aid in identifying the compounds based upon standardized retention and aroma.

### Statistical analysis

We conducted similar experiments on unpanned oolong tea with three replications and the results from GC‐MS and GC‐O were compared with the panned tea from this study. The data from GC‐MS were analyzed by JMP 11.0 (SAS, Cary, NC). Two way analysis of variance (ANOVA) and mean comparisons using Tukey's test with the 5% significance level were conducted on different compound categories: alcohols, aldehydes, ketones, terpenes, acids and ester results from SDE and SPME techniques of panned and unpanned teas.

One way ANOVA was also used to find significant differences in 12 volatiles (previously reported to be major flavor compounds in oolong tea) extracted with SDE and SPME techniques in panned and unpanned tea. Means were compared by using Fisher's least significant difference (LSD) method with significance at *P* < 0.05.

## Results and Discussion

### GC‐MS analysis

A total of 190 volatile compounds were identified using SDE and SPME with GC‐MS and GC‐O. We identified 200 volatile compounds in unpanned oolong tea, of which only 79 of these compounds were found in panned oolong tea (Table 1); this shows the significant impact of panning on flavor volatiles of oolong tea. We also observed that the compounds identified using SDE and SPME differed and were complementary. Therefore, we used the same approach to analyze and discuss our results from GC‐MS and GC‐O.

**Table 1:** Identified volatiles in the panned oolong tea with method of identification, LRI and comparisons with the unpanned tea

No.	Compound	LRI	SDE	SPME	Unpanned
1	Butanenitrile, 2‐methyl‐	637	MS		
2	Butanenitrile, 3‐methyl‐	646	MS		X
3	2H‐Pyran, 3,4‐dihydro‐6‐methyl‐	644	MS		
4	Pyrrole	646	MS		
5	1‐Pentanol	657	MS		X
6	Pentane, 1‐chloro‐	662	MS		
7	2‐Penten‐1‐ol, (Z)‐	661	MS		X
8	Cyclopropane, 1,1,2,3‐tetramethyl‐	695	MS		
9	Hexanal	700		MS	X
10	3(2H)‐Furanone, dihydro‐2‐methyl‐	703	MS		X
11	1H‐Pyrrole, 1‐ethyl‐	711	MS	MS	
12	Pyrazine, methyl‐	717	MS	MS	
13	Maleic anhydride	724	MS		
14	Furfural	727	MS	MS	X
15	2‐Hexenal, (E)‐	747	MS		X
16	2‐Furranmethanol	750	MS	MS	
17	p‐Xylene	764	MS		
18	Benzene, 1,3‐dimethyl‐	767		MS	
19	1,5‐Heptadiene, 2,6‐dimethyl‐	781	MS		
20	Oxime‐, methoxy‐phenyl‐_	802	MS		X
21	Ethanone, 1‐(2‐furanyl)‐	808	MS		
22	1‐(3H‐Imidazol‐4‐yl)‐ethanone	810		MS	
23	Pyrazine, ethyl‐	811	MS		
24	1H‐Pyrrole‐2‐carboxaldehyde, 1‐methyl‐	824	MS	MS	
25	Benzaldehyde	857	MS	MS	X
26	2‐Furancarboxaldehyde, 5‐methyl‐	860	MS	MS	
27	Methyl 2‐furoate	873	MS		
28	1‐Octen‐3‐ol	877	MS		X
29	Sulcatone	884	MS		X
30	*β*‐Myrcene	889	MS	MS	
31	Pyrazine, 2‐ethyl‐6‐methyl‐	895	MS		X
32	2,4‐Hexadienal	928			
33	Bicyclo[2.2.1]heptane, 2‐butyl‐	909	MS		
34	Furan, 2‐propyl‐	910	MS		
35	1,3‐Cyclohexadiene, 1‐methyl‐4‐(1‐methylethyl)‐	920	MS		
36	Mesitylene	930	MS		X
37	o‐Cymene	931	MS	MS	X
38	D‐Limonene	938	MS	MS	X
39	Benzyl alcohol	944	MS		X
40	trans‐*β*‐Ocimene	951	MS	MS	X
41	Benzeneacetaldehyde	957	MS	MS	X
42	1H‐Pyrrole‐2‐carboxaldehyde, 1‐ethyl‐	965	MS	MS	X
43	Cyclohexene, 1‐(3‐ethoxy‐1‐propenyl)‐, (Z)‐	973	MS		
44	Ethanone, 1‐(1H‐pyrrol‐2‐yl)‐	980	MS	MS	
45	Acetophenone	989	MS		
46	1‐Octanol	997	MS		
47	Linalool oxide	1000	MS	MS	X
48	Pyrazine, 3‐ethyl‐2,5‐dimethyl‐	1019	MS		
49	Linalool oxide(furanoid)	1053	MS		X
50	3,5‐Octadien‐2‐one	1066	MS		X
51	R‐Linalool	1091	MS	MS	X
52	Hotrienol	1102	MS	MS	
53	3,4‐Dimethylcyclohexanol	1103		MS	X
54	Benzenamine, 4‐methoxy‐2‐methyl‐	1110	MS	MS	
55	1,5,9‐Undecatriene, 2,6,10‐trimethyl‐, (Z)‐	1115	MS		
56	Isophorone	1118	MS		X
57	2,4,6‐Octatriene, 2,6‐dimethyl‐, (E,Z)‐ E,Z‐Alloocimene	1128	MS		
58	Benzyl nitrile	1136	MS	MS	X
59	1,3‐Cyclopentadiene, 1,2,3,4‐tetramethyl‐5‐methylene‐	1151	MS		
60	1‐[2‐Aminoethyl]hypoxanthine	1154	MS		
61	2‐Nonenal, (E)‐	1158	MS		
62	1H‐Pyrrole‐3‐carboxylic acid, 2,4‐dimethyl‐, methyl ester	1161	MS		
63	Benzeneacetic acid,.*α*.‐oxo‐, ethyl ester	1163	MS		
64	2H‐Pyran‐3‐ol, 6‐ethenyltetrahydro‐2,2,6‐trimethyl‐	1168	MS		X
65	Benzeneacetic acid, methyl ester	1176	MS		
66	3‐Amino‐4‐methylbenzyl alcohol	1181	MS	MS	
67	Butanoic acid, 3‐hexenyl ester, (E)‐	1184	MS		
68	*α*‐Terpineol	1190	MS	MS	X
69	Methyl salicylate	1193	MS	MS	X
70	1,3‐Cyclohexadiene‐1‐carboxaldehyde, 2,6,6‐trimethyl‐	1199	MS		
71	Decanal	1202		MS	X
72	(Z)‐4‐Decenal	1203			
73	1H‐Indene, 2,3‐dihydro‐1,1,5,6‐tetramethyl‐	1206	MS		
74	Benzene, (ethenyloxy)‐	1208	MS		
75	1,3‐Cyclohexadiene‐1‐methanol, 4‐(1‐methylethyl)‐	1211	MS		
76	*β*‐Cyclocitral	1211	MS		
77	4a(2H)‐Naphthalenol, octahydro‐, trans‐	1213	MS		
78	Benzene, 1‐(1,5‐dimethylhexyl)‐4‐methyl‐	1216	MS		X
79	Prop‐2‐en‐1‐one, 1‐(6,6‐dimethylbicyclo[3.1.1]hept‐2‐en‐2‐yl)‐	1218	MS		
80	Citral	1221	MS		X
81	Geraniol	1227	MS	MS	X
82	Acetic acid, 2‐phenylethyl ester	1229	MS	MS	X
83	Isocyclocitral	1230	MS		
84	Nonanoic acid	1234		MS	X
85	2,6‐Octadienal, 3,7‐dimethyl‐, (E)‐	1236	MS		
86	2(1H)‐Naphthalenone, 3,4,4a,5,6,7‐hexahydro‐1,1,4a‐trimethyl‐	1242	MS		
87	Ionone	1242	MS	MS	X
88	4‐Acetamido‐2‐methallylphenol	1244	MS		
89	Indole	1248	MS		X
90	Formic acid, (2‐methylphenyl)methyl ester	1250	MS		X
91	Pyrazine, 2,5‐dimethyl‐3‐propyl‐	1253	MS		
92	Cyclohexane, 1,2‐diethenyl‐4‐(1‐methylethylidene)‐, cis‐	1256	MS		
93	4‐Hydroxy‐3‐methylacetophenone	1258	MS		
94	Spiro[3.6]deca‐5,7‐dien‐1‐one,5,9,9‐trimethyl	1259	MS	MS	
95	2H‐Pyran‐3‐ol, 2‐ethoxy‐3,4‐dihydro‐, acetate	1262		MS	
96	2,6‐Octadienoic acid, 3,7‐dimethyl‐, methyl ester	1263	MS		
97	6‐Hydroxynicotinic acid di‐methyl derivative	1264	MS		
98	Pentanoic acid, 4‐methyl‐, ethyl ester	1270		MS	
99	Benzene, 2‐(2‐butenyl)‐1,3,5‐trimethyl‐	1277	MS		
100	1, 1, 5‐Trimethyl‐1, 2‐dihydronaphthalene	1278	MS	MS	
101	Naphthalene, 1,2,3,4‐tetrahydro‐1,1,6‐trimethyl‐	1280	MS		
102	Bicyclo[3.1.0]hexan‐3‐ol, 4‐methyl‐1‐(1 methylethyl)‐	1282	MS		
103	Phenol, 2‐(1,1‐dimethyl‐2‐propenyl)‐3,6‐dimethyl‐	1284	MS		
104	cis‐anti‐cis‐Tricyclo[7.3.0.0(2,6)]‐7‐dodecene	1286	MS		
105	Hexanoic acid, hexyl ester	1292	MS		
106	Decanoic acid, ethyl ester	1297	MS	MS	X
107	*cis*‐Jasmone	1299	MS	MS	X
108	Naphthalene, 1,2,3,4‐tetrahydro‐2,5,8‐trimethyl‐	1402	MS		
109	Cyclopropanecarboxylic acid, 2,2‐dimethyl‐3‐(2‐methyl‐1‐propenyl)‐, 2‐methyl‐4‐oxo‐3‐(2‐pentenyl)‐2‐cyclopenten‐1‐yl ester, [1R	1423	MS		
110	*α*‐Ionone	1428	MS		
111	6,7‐Dimethyl‐1,2,3,5,8,8a‐Hexahydronaphthalene	1432	MS		
112	Coumarin	1437	MS		X
113	*β*‐Phenylethyl butyrate	1440	MS		X
114	(E)‐Geranyl acetone)	1451	MS		X
115	cis‐ *β*‐Farnesene	1456	MS		X
116	4‐(2,4,4‐Trimethyl‐cyclohexa‐1,5‐dienyl)‐but‐3‐en‐2‐one	1484	MS		
117	*trans*‐. *β*.‐Ionone	1488	MS		X
118	Jasmin lactone	1493	MS	MS	X
119	1H‐Benzocyclohepten‐7‐ol, 2,3,4,4a,5,6,7,8‐Octahydro‐1,1,4a,7‐tetramethyl‐, cis‐	1499	MS		
120	Gamma.‐Muurolene	1504	MS		
121	*α*‐Farnesene	1508	MS	MS	X
122	Bicyclo[2.2.1]heptan‐2‐one, 1‐(bromomethyl)‐7,7‐dimethyl‐, (1S)‐	1512	MS		X
123	Butylated Hydroxytoluene	1514	MS		X
124	cis‐Thujopsene	1519	MS		X
125	Levomenol	1545	MS		
126	trans‐Nerolidol	1564	MS	MS	X
127	3‐Hexen‐1‐ol, benzoate, (Z)‐	1572	MS		X
128	Benzoic acid, hexyl ester	1579	MS		
129	Farnesene epoxide, E‐	1599	MS		X
130	Cyclopentaneacetic acid, 3‐oxo‐2‐(2‐pentenyl)‐, Methyl ester, [1.*α*.,2.*α*.(Z)]‐	1649	MS		
131	2‐Furanmethanol, tetrahydro‐.*α*.,.*α*.,5‐trimethyl‐5‐(4‐methyl‐3‐cyclohexen‐1‐yl)‐, [2S‐[2.*α*.,5. *β*.(R*)]]‐	1660	MS		
132	Phytol	1836	MS		

A total of 121 volatiles were extracted from panned oolong tea using SDE. Among these compounds, 18 alcohols, 11 aldehydes, 16 ketones, 23 terpenes and 13 acids were identified. The most abundant compounds were furfural (10.8%), *trans*‐nerolidol (8.5%), *α*‐farnesene (4.8%), ethyl‐pyrrole‐2‐carboxaldehyde (3.9%), benzyl nitrile (3.5%), 5‐methyl‐2‐furancarboxaldehyde (2.8%), indole (2.5%), 4‐methoxy‐2‐methyl‐benzenamine (2.3), 3‐methyl‐butanenitrile (2.1%) and 1‐(2‐furanyl)‐ethanone (2.1%). Only *trans*‐nerolidol, *α*‐farnesene, indole and benzyl nitrile also appeared as most abundant compounds in unpanned tea. Similar to unpanned tea, *trans*‐ nerolidol (43.9% of total alcohols) and indole (13.4%) were the two major alcohols. Major ketones were 1‐(2‐furanyl)‐ethanone (21.5% of total ketones) and dihydro‐2‐methyl‐3(2H)‐furanone (11.0%); however, the major ketones in unpanned tea were jasmine lactones and *trans*‐*β*‐ionone. These two compounds were identified in panned tea, but at much lower concentrations. Furfural (49.5% of total aldehydes) was the most abundant aldehyde in panned tea, but hexanal and benzeneacetaldehyde were the most abundant aldehydes in unpanned tea. Among 23 identified terpenes in panned tea, *α*‐farnesene (24.2%) and linalool oxide (9.1%) were the compounds with highest peak areas. For unpanned tea, *α*‐farnesene was the most abundant terpene, followed by geraniol and linalool. Sesquiterpenes in oolong tea may be present as glucosides that can be hydrolyzed to form various aromatic compounds during the manufacturing process (Guo et al. 1996). These glucosides may also be obtained by biosynthesis during the manufacturing process (Wang et al. 2001).

A total of 48 volatile compounds were detected using SPME, including eight alcohols, eight aldehydes, four ketones, seven acids, and 11 terpenes. The compounds with highest peak area were indole (9.3%), furfural (7.1%), 1‐ethyl‐1‐pyrrole‐2‐carboxaldehyde (5.5%), benzyl nitrile (4.1%), 1,1,5‐trimethyl‐1,2‐dihydronaphthalene (TDN) (3.4%), 4‐methoxy‐2‐methyl‐benzenamine (2.9%), 5,9,9‐trimethyl‐spiro[3.6]deca‐5,7‐dien‐1‐one (2.8%), methoxy‐phenyloxime (2.5%), 3,4‐dimethylcyclohexanol (2.4%), and 3‐amino‐4‐methylbenzyl alcohol (2.4%). Indole (44.7% of total alcohols) was the most abundant alcohol for panned and unpanned teas. Similar to what was observed for SDE, furfural (31.6% of total aldehydes) had the highest peak area in panned tea, but for unpanned tea the most abundant aldehyde was 2,4‐decadienal. Hotrienol (18.5% of total terpenes) had the highest peak areas among terpenes, but this compound was not identified in unpanned tea. The most abundant terpene in the unpanned tea was geraniol.

**Figure 1:**
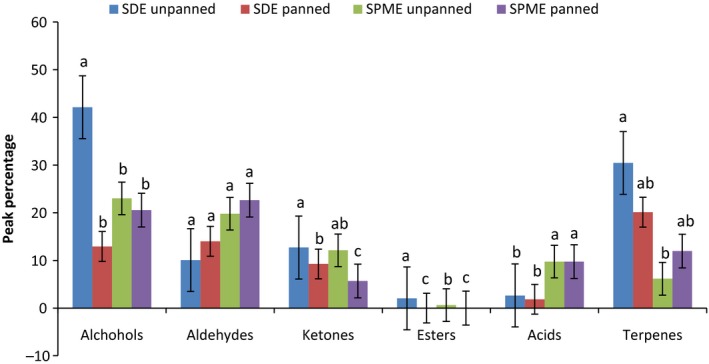
Mean comparison of peak percentages of chemical composition the unpanned and panned tea using SDE and SPME. Means within a class of compounds with the same letter are not significantly different (*P* > 0.05). Bars represent standard deviations.

Results from ANOVA showed there was a significant difference (*P* < 0.05) between the summed peak percentages of alcohols between panned and unpanned teas (Fig. 1). The percentage of alcohols in unpanned tea were significantly higher (*P* < 0.05) than panned tea from SDE; however, there was no significant difference (*P* > 0.05) in alcohols percentages between unpanned and panned tea with SPME. Fermentation results in increased enzyme activity that leads to creation of aromatic alcohols (Ma et al. 2014); however, during panning, many of these enzymes are destroyed by heat, which results in less formation of aromatic alcohols. There were no significant differences (*P* > 0.05) in aldehyde percentages of panned and unpanned tea in both extraction techniques. Analysis of ketones showed significant differences (*P* < 0.05) in panned and unpanned tea in both extraction techniques. Additionally, the peak percentages of panned tea for ketones were higher (*P* < 0.05) in SDE compared to SPME. No esters were identified in panned tea. There were no differences (*P* > 0.05) between the acids contents of panned and unpanned tea in both SDE and SPME. The percentages of terpenes in both extraction techniques were not different (*P* > 0.05) between panned and unpanned tea; however, terpene percentages of unpanned tea in SDE were higher (*P* < 0.05) than for SPME.

Most of the published studies on oolong tea volatiles have either investigated major compounds that differentiate oolong with fully fermented teas or nonfermented teas or have studied the compounds that are indicator of quality in oolong tea. During fermentation, several enzymatic reactions are responsible for formation of tea aroma compounds. The main precursors for tea aroma are amino acids and carotenoids, including *β*‐carotene, lutein, neoxanthin, and violaxanthin (Yamanishi 1978). During fermentation, oxidation results in the significant reduction of carotenoids, particularly *β* carotene, resulting in the formation of ionone and terpenoid carbonyls (Yamanishi 1978). After oxidation and secondary epoxidation reactions, other carotenoids give rise to ionone, linalool and substituted hydroxy‐ and epoxy‐ionones (Sanderson and Grahamm 1973). Generally, grassy or green flavors are diminished during fermentation, but fruity, floral and other fermented characters are increased (Wang et al. 2008).

Pripdeevech and Machan (2011) used *cis*‐jasmone (woody, herbal), *trans*‐nerolidol (floral), indole (pungent) and hotrienol to differentiate semifermented tea from nonfermented tea. They showed the content of the first three volatiles were increased significantly while hotrienol (green, sweet) was decreased after fermentation. In our study, the content of *cis*‐jasmone was significantly higher (*P* < 0.05) in panned tea while the content of indole and *trans*‐nerodiol were significantly decreased (*P* < 0.05) by panning. The content of indole is very low in nonfermented tea, but its level increases quickly at the beginning of fermentation in oolong tea and then slowly decreased by continuing fermentation (Wang et al. 2008). Indole precursors might be destroyed by the heat treatment during panning and lead to changes in indole contents in panned tea. GC‐MS analysis was able to identify hotrienol only in the panned tea, suggesting perhaps that heat treatment resulted in formation of hotrienol in oolong tea.

Other studies showed other compounds may be important in distinguishing oolong from other varieties of teas. Other than indole, Wang et al. (2008) found flavor compounds such as *trans*‐2‐hexenal (green), benzaldehyde (almond), and methyl salicylate (peppermint) are important to distinguish unfermented teas from fermented ones. *Trans*‐2‐hexenal and methyl salicylate also may be used to classify the semi from fully fermented teas. Others have also reported the contents of compounds such as (E)‐geraniol (floral, rose), (E)‐*β*‐damascenone (not identified in our study), and linalool oxide B (floral) increase with degrees of fermentation (Kawakami et al. 1995; Wang et al. 2008, 2011; Zhang et al. 2013). In our study, *trans*‐2‐hexenal, methyl salicylate and geraniol contents were decreased significantly (*P* < 0.05) by panning, but linalool oxide content was significantly (*P* < 0.05) increased. *Trans*‐2‐hexenol (grassy, green) is a product of lipid degradation and result in inferior quality to tea (Pripdeevech and Machan 2011). Usually, a higher amount of *trans*‐2‐hexenal is detected in nonfermented tea whereas in semifermented tea concentrations are reportedly significantly lower (0.04–0.08%) (Pripdeevech and Machan 2011). Our results suggest that panning in oolong tea promotes formation of some of the flavor characteristics of nonfermented as well as fermented teas.

**Figure 2:**
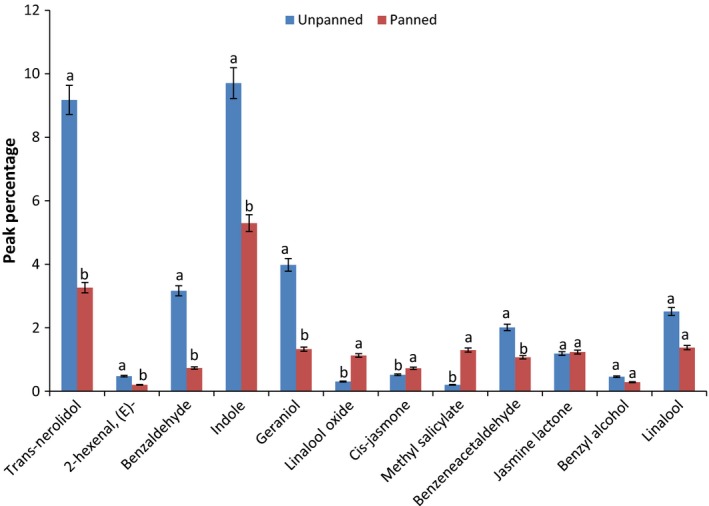
Mean comparison of peak percentage of 12 major volatiles in the panned and unpanned tea identified from SDE and SPME techniques. Means with the same letter within each compounds are not significantly different (*P* > 0.05). Bars represent standard deviations.

Several studies suggest that volatile flavor compounds affect the perceived quality of oolong tea. *Trans*‐nerolidol was reported as one of the key odorants and can be considered as an indicator for the high quality oolong tea flavor (Kai et al. 2008; Pripdeevech and Machan 2011; Wang et al. 2011; Zou et al. 2011; Ma et al. 2014). We found that *trans*‐ nerolidol was the most dominant volatile in unpanned oolong tea; however, during panning the concentration of these compound significantly decreased (*P* < 0.05) (Fig. 2). Similar to our results, Ma et al. (2014) reported decreases in the nerolidol content during other thermal process steps in oolong tea manufacturing such as fixation, shaping, and drying. Nerolidol has a floral aroma (Lapczynski et al. 2008) and exists at a relatively high concentration in oolong tea. Even though the concentration of this compound was significantly decreased by panning, it was the second most abundant compound in the panned tea in our study. The content of nerolidol is low in fresh leaves, but the content was greatly increased and reaches to its highest level during the fermentation stage of manufacturing (Ma et al. 2014).

Wang et al. (2011) reported that perceived aroma score positively correlated with concentrations of benzyl alcohol (sweet, flower), benzeneacetaldehyde (honey, floral), linalool (flower), phenylethyl alcohol (honey), linalool oxide (flower), indole (pungent), *cis*‐jasmone (herbal, woody), nerolidol (flower), and methyl jasmonate (flower). In addition, they found the total quality score positively correlated with concentrations of benzyl alcohol, benzeneacetaldehyde, geraniol, indole and toluene (not identified in our study), but negatively correlated with the concentrations of (E,E)‐2,4‐heptadienal (identified by GC‐O in our study). Other studies also showed benzaldehyde (almond) (Zhang et al. 2013), jasmine lactone (floral and fruity) (Wang et al. 2001, 2008, 2011; Zhang et al. 2013), and *α*‐farnesene (woody) (Kawakami et al. 1995; Wang et al. 2008, 2011; Zhang et al. 2013) play important roles in aroma of oolong tea and have high correlation with the aroma of quality oolong tea. Compounds such as (E)‐*β*‐damascenone (Zhang et al. 2013) and 5‐methyl‐hepten‐2‐one (Wang et al. 2008) are also reported as major flavor compounds, but they were not detected in our study. In addition, phenylethyl alcohol was identified in high concentration in the unpanned tea but was not detected in the panned tea. There were significant reductions (*P* < 0.05) in amounts of *trans*‐nerolidol, 2‐hexenal, benzaldehyde, indole, geraniol, and benzenacetaldehyde as a result of panning. However, panning caused significant increases (*P* < 0.05) in contents of linalool oxide, *cis*‐jasmone, and methyl salicylate. There were no significant differences (*P* > 0.05) in content of linalool, jasmine lactone and benzyl alcohol between panned and unpanned tea.

Furfural was the most dominant volatile in the panned tea analysed using SDE extraction and was also identified as the second most abundant compound using SPME. Furfural was not identified in unpanned tea with either extraction technique using GC‐MS analysis, but we did identify it as aroma active compound with GC‐O. Furfural has been reported in flavor profile of oolong tea in previous studies (Wang et al. 2008; Pripdeevech and Machan 2011; Zhang et al. 2013). Furfural has been found in an extensive range of teas, coffees, fruits, and wine and has been used as an ingredient for flavor enhancements in food (Rega et al. 2009). Furfural odor is like baked bread, almond and sweet (Rega et al. 2009). Furfural is formed by the heat treatment or acid hydrolysis of polysaccharides, which contain hexose and pentose fragments (IARC 1995). Panning can create compounds that are generated in the thermal degradation of cellulose and hemicellulose such as furfurals, 5‐methyl‐2‐furancarboxaldehyde and other furans (Guillén and Manzanos 1997; Sung 2013).

Pripdeevech and Machan (2011) also indicated that the process of steaming or panning at high temperature in nonfermented tea method may produce lipid degradation products such as heptanoic acid, 2,6,6‐trimethyl‐1‐cyclohexene‐1‐carboxaldehyde, or nonanoic acid. In comparison to unpanned tea, many pyrrole compounds were identified in the panned tea such as 1H‐pyrrole,1‐ethyl‐pyrrole, 1‐methyl‐1H‐pyrrole‐2‐carboxaldehyde, 1‐(1H‐pyrrol‐2‐yl)‐ethanone, 2‐acetylpyrrole, and 2,4‐dimethyl‐1H‐pyrrole‐3‐carboxylic acid methyl ester. The generation of these nitrogen‐containing heterocyclic compounds is assumed to be caused by the Strecker degradation of theanine and amino acids during the tea preparation, and is responsible for the aroma of the heat treated teas (Yu et al. 1999).

### GC‐O analysis

In SDE extraction, 47 aroma active volatiles were tentatively identified based on the combination of LRI and odor descriptors in panned oolong tea and nine of these compounds were also identified by GC‐MS analysis (Table 2). Among these compounds, 17 compounds were previously identified by both extraction techniques in the unpanned tea. However, in SPME, we identified 42 compounds that possessed aroma activity, but only 10 of these compounds were identical to extracted aroma compounds from SDE (Table 3). Among these 42 compounds, 17 compounds were shared with unpanned tea and only six of these 42 compounds were detected by GC‐MS analysis.

**Table 2:** The aroma active compounds in the panned oolong tea using time‐intensity GC‐O with SDE extraction

No.	Compound	LRI	Confirmed^c^	Aroma Description	Intensity^d^
1	n.i.^e^	561–580		Nutty, Chocolate, caramel	4
2	Methylbutenol	620	620	Green, herb	2
3	Methylbutanal^a^^,^ b	647	641	Popcorn, nutty, chemical	2
4	Isobutyraldehyde	655	662	Green	2
5	Methyl methylbutanoate	675	674	Fruity	2
6	Pentenone^a^^,^ b	682	780	Fruity	2
7	*α*,*γ*‐Dimethylallyl alcohol	702	712	Green, sweet, fruit	3
8	Methylcyclohexane	715–723	716^b^	Sweet, nutty, cookies	1
9	Methyl butanoate^a^^,^ b	727	723	Sweet	3
10	Pentanal^a^^,^ b	740	732	Smoky, nutty	4
11	Methyl‐2‐butenal^b^	747	752	Green pepper	1
12	Methyl‐2‐butenol^a^	774	775	Celery, herb	2
13	3‐Hexenal	798	800	Green, earthy	2
14	**Furfural** a ^,^ b	825	829	Nutty, chocolate	2
15	n.i.^a^^,^ e	830–837		Waxy, smoky	1
16	**(E)‐2‐Hexenal**	851	844	Fruity	2
17	Furfuryl alcohol	855	851	Smoky, burnt	1
18	2‐Hexenal^b^	859–865	854	Fruity	2
19	Heptanal	882	885^b^	Burnt plastic, smoky	2
20	**Benzaldehyde** a	964	960	Nutty	2
21	**(E)‐2‐Penten‐1‐ol**	965	973	Mushroom	2
22	Filbertone	972	972	Nutty	2
23	**6,6‐Dimethyl‐2‐methylenebicyclo[3.1.1]heptane**	984	981^b^	Nutty, musty, sweet	2
24	Acetylthiazole	1017–1024	1020	Nutty, Waxy	2
25	1,8‐Cineole	1025–1029	1032	Nutty, floral, sweet	2
26	(E)‐2‐Heptenal	1041	1041	Waxy, fatty	3
28	2‐Octenal	1055	1060^b^	Nutty,	2
29	*α*‐Ocimene^b^	1058	1056	Fruity, floral	1
30	p‐Cresol	1076	1075	Smoky	1
31	3,5‐Octadienone	1096	1095	Citrus, fruity, sweet	2
32	**(E)‐Linalool oxide**	1176	1172	Sweet, floral, fruity	2
33	Isobutylmethoxypyrazine	1189	1186	Green, smoky	2
34	Decanal	1211	1211^b^	Waxy	3
35	**Linalool oxide** a ^,^ b	1217	1212^b^	Floral, fruity, earthy	2
36	Nerol^b^	1239	1233	Sweet	2
37	Benzothiazole^a^	1246	1240	Smoky	3
38	Isobornyl formate	1249	1245	Earthy	2
39	Dihydromethylcyclopentapyrazine^a^	1255	1248	Nutty, smoky	4
40	Linalyl acetate^a^	1264–1271	1261	Sweet, floral	1
41	Safrole^a^	1281	1280	Spicey, smoky	2
42	cis‐Linalool pyran oxide	1401	1402	Citrus	2
43	n.i.^e^	1417–1421		Smoky, cooked meat	4
44	**Coumarin**	1433	1439	Sweet, waxy	3
45	Linalyl isovalerate	1477	1478	Fruity, waxy	2
46	Citronellyl butyrate	1536	1528	Fruity	3
47	**(Z)‐Nerolidol**	1570	1565	Waxy	3

Bold Compounds were also detected with GC‐MS.

a Identified with SDE in GC‐O analysis of unpanned tea.

b Identified with SPME in GC‐O analysis of unpanned tea.

c LRI values confirmed with databases Flavornet and Pherobase to identify the compounds based upon standardized retention and aroma.

d The average aroma intensity score by panelist on a scale of 5 where 1 = low intensity and 5 = high intensity.

e Not identified compound.

**Table 3:** The aroma active compounds in the panned oolong tea using time‐intensity GC‐O with SPME extraction

No.	Compound	LRI	Confirmed^c^	Aroma description	Intensity^d^
1	Methylbutenol	609	620	Spice, herb	1
2	Methylbutanal^a^	654	641	Sweet, vanilla, almond	1
3	Methyl 2‐methylpropionate	687–688	685^b^	Fruity, sweet	2
4	*α*,*γ*‐Dimethylallyl alcohol	718	712	Green	1
5	Methyl butanoate^a^^,^ b	723	723	Fruity, sweet	3
6	Pentanal^a^^,^ b	728	732	Nutty	1
7	Pentanol^a^	765	759	Fruity	2
8	Methyl‐2‐butenol	788	779	Spicy, herb	1
9	1‐Hexenol	792	789	Green pepper	3
10	n.i.^e^	800–807	‐	Smoky, earthy	3
11	Propyl propanoate^a^	817	812	Sweet	1
12	**Furfura**l^a^^,^ b	829	829	Nutty, earthy	2
13	**Methyl pyrazine**	833–835	828	Nutty, popcorn	1
14	n.i.^b^^,^ e	839–846	‐	Chocolate, nutty	1
15	Isopropyl butanoate^a^	854	847	Fruity, floral	2
16	(Z)‐3‐hexenol	861	858	Green	1
17	2‐Methylbutyl acetate^b^	883–885	880	Sweet, fruit	3
18	2,4‐Hexadienal	906	910	Grassy	2
19	Dimethylthiazole^a^^,^ b	928	928	Plastic, smoky	1
20	Heptanol^b^	970	962	Green	2
21	Ethyl isohexanoate^b^	971	968	Fruity	2
22	1,5‐Octadienone	983	988	Musty	1
23	Ethylmethyl pyrazine^a^^,^ b	986	993	Fruity	2
24	2,4‐Heptadienal^b^	1019–1023	1011	Nutty, sweet	1
25	**(E)‐** *β* **‐Ocimene**	1038	1038	Sweet, vanilla	3
26	2‐Acetylpyrrole	1051	1045	Nutty	1
27	*α*‐Ocimene	1062	1056	Apple, banana, fruity	3
28	Ethyldimethylthiazole	1073	1078	Earthy	1
29	Limonene oxide	1129	1132	Fruity	4
30	*γ*‐Heptalactone	1130	1130	Nutty	2
31	3,5‐Diethyl‐2‐methylpyrazine^a^	1160	1160^b^	Nutty, chocolate	1
32	Dihydrocarveol	1191	1190	Spicey, mint	1
33	Ethyl octanoate	1197	1198	Floral, fruity	3
34	(Z)‐4‐Decenal	1203	1200	Green, musty	1
35	**Linalool oxide** b	1214	1212	Green, floral	1
36	3‐Phenylpropan‐1‐ol	1220	1219 b	Fruity	1
37	**5‐Methyl‐2‐furancarboxaldehyde**	1222	1224^b^	Musty	1
38	Nerol	1226	1233	Sweet	1
39	**Methyl salicylate**	1234	1234	Nutty, floral	2
40	Isobornyl formate	1246	1245	Green	2
41	n.i.^e^	1252–1261	‐	Nutty, chocolate	3
42	Linalyl acetate^b^	1263	1261	Sweet	2

Bold Compounds were also detected with GC‐MS.

a Identified with SPME in GC‐O analysis of unpanned tea.

b Identified with SDEE in GC‐O analysis of unpanned tea.

c LRI values confirmed with databases Flavornet and Pherobase to identify the compounds based upon standardized retention and aroma.

d The average aroma intensity score by panelist on a scale of 5 where 1 =  low intensity and 5 =  high intensity.

e Not identified compound.

**Figure 3:**
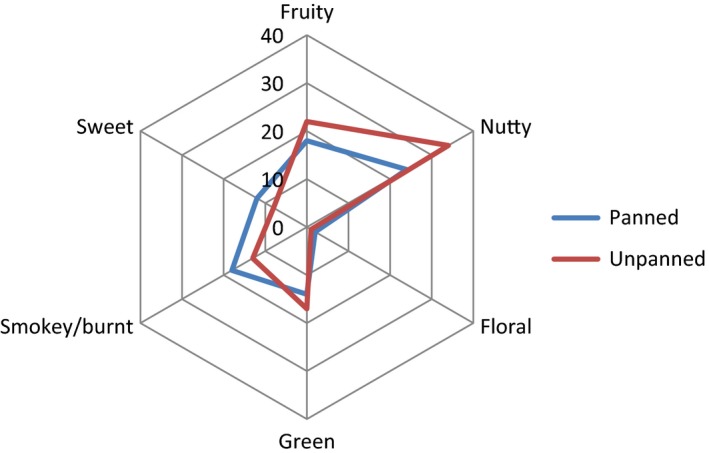
Radargram of aroma profile of panned oolong tea using SDE obtained from grouping of identified compounds using GC‐O with similar aroma characteristics.

**Figure 4:**
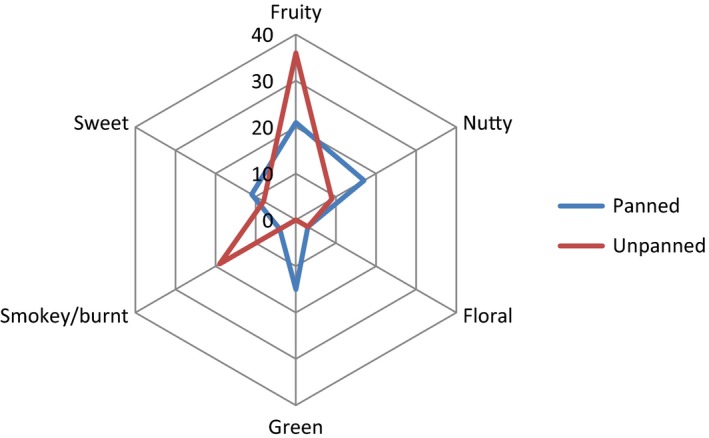
Radargram of aroma profile of panned oolong tea using SPME obtained from grouping of identified compounds using GC‐O with similar aroma characteristics.

The identified aroma components of panned and unpanned tea for both extraction techniques were grouped in six categories based on their aroma description: fruity, sweet, floral, nutty, green, and smoky/burnt. The total aroma intensities of identified aroma compounds from SDE and SPME for each aroma group are shown in Figs. 3 and 4 respectively. The most important features that were consistent between these two Figures were the increase of sweet aroma and decrease of fruity and smoky in the panned tea. Floral aroma was not considerably affected by panning. Green aroma was the most different feature between two Figures. Previously, our panelists were unable to detect any green aroma in unpanned tea from SPME extraction; however, for the panned tea, a number of compounds responsible for green and grassy flavor were identified. On the other hand, with SDE the green aroma was slightly decreased. The other inconsistent result was related to nutty aroma. In SDE, there were more intense aroma active compounds with nutty smells detected in the unpanned tea. In contrast with SDE results, total aroma intensity for nutty smell was increased by panning which is more consistent with the literature. The differences in two methods' capabilities have been indicated in some other flavor studies. A SPME extraction technique was reportedly unsuitable for the isolation of high molecular compounds or for those with a strong affinity to the matrix; however, some compounds cannot be detected in SDE due to the presence of solvent (Majcher and Jelenń 2009), and also some artificial compounds can be generated during the extraction.

Among the identified aroma active compounds in the panned tea using SDE, pentanal (smoky), dihydromethylcyclopentapyrazine (nutty), and one unidentified compound (nutty) had the highest aroma intensity in the panned tea. Dihydromethylcyclopentapyrazine and the unidentified compound had nutty flavors and were also detected among the most intense aroma compounds in the unpanned tea. However, limonene oxide (fruity) was the only compound that scored 4 in SPME. We were able to identify (E, E)‐2, 4‐heptadienal (nutty) and (Z)‐3‐Hexenol (green) in our GC‐O analyses, which have been shown to be increased by degree of fermentation (Wang et al. 2008, 2011). Similar to the unpanned tea, *trans*‐2‐hexenol was identified, which is considered an off‐flavor in oolong tea. We also detected pyrazines such as ethylmethyl pyrazine and dihydromethylcyclopentapyrazine, which are known as thermally generated product of amino acids and sugars (Kato and Shibamoto 2001; Wang et al. 2010); however, both of these compounds were found in unpanned tea as well. Jin Xuan oolong is well‐known for its milky aroma. Previously, we identified dieactyl with butter aroma and suggested that the milky aroma of this variety of tea is associated with this compound. However, this compound was not detected by our panelists in the unpanned tea and panning might have resulted in elimination of the milky aroma in Jin Xuan oolong.

## Conclusions

Despite a few similarities in the most abundant identified compounds from GC‐MS analysis and aroma active compounds from GC‐O analysis between the unpanned and panned tea, panning significantly changed the aroma volatile components of oolong tea. The abundance of alcohols, ketones, acids and esters were significantly changed by panning; however, there were no changes in contents of terpenes and aldehydes. Since over‐heating/panning the leaves may result in a burnt odor and underpanning may result in a greenish odor and red central vein (Hui et al. 2003), optimization of time and temperature in panning to manufacture best quality tea need to be investigated for the future studies. Moreover, conducting sensory studies to better understand consumer perception of panning effects on quality of oolong tea is necessary for large‐scale manufacturing and commercialization of panned tea.

## Conflict of Interest

None declared.
